# Prevalence of Medication-Dietary Supplement Combined Use and Associated Factors

**DOI:** 10.3390/nu11102466

**Published:** 2019-10-15

**Authors:** Ignacio Aznar-Lou, Cristina Carbonell-Duacastella, Ana Rodriguez, Inés Mera, Maria Rubio-Valera

**Affiliations:** 1 Teaching, Research & Innovation Unit, Institut de Recerca Sant Joan de Déu, Esplugues de Llobregat, Parc Sanitari Sant Joan de Déu, 08830 Sant Boi de Llobregat (Barcelona), Spain; c.carbonell@pssjd.org (C.C.-D.); mrubio@pssjd.org (M.R.-V.); 2 Centro de Investigación Biomédica en Red en Epidemiología y Salud Pública, CIBERESP, 28029 Madrid, Spain; 3 Spanish Society of Community and Family Pharmacy (SEFAC), 28045 Madrid, Spain; ana.rodriguez@sefac.org (A.R.); ines.mera@hotmail.es (I.M.); 4 School of Pharmacy, University of Barcelona, 08028 Barcelona, Spain

**Keywords:** prevalence, interactions, dietary supplements, antibiotics, antihypertensive medication

## Abstract

Introduction: The use of medication has increased in recent years in the US while the use of dietary supplements has remained stable but high. Interactions between these two kinds of products may have important consequences, especially in the case of widely used medications such as antihypertensives and antibiotics. The aim of this paper is to estimate the prevalence of potentially serious drug–dietary supplement interactions among tetracyclines, thiazides, and angiotensin II receptor blocker users by means of the NHANES 2013–2014 dataset. Methods: Data from 2013–2014 NHANES were obtained. Potential interactions analysed were tetracyclines with calcium, magnesium, and zinc, thiazides with vitamin D, and angiotensin II receptors blockers with potassium. Prevalence was calculated for each potential interaction. Logistic regression was used to assess associated factors. Results: 864 prescriptions issued to 820 patients were analysed. Overall prevalence of potential interaction was 49%. Older age and higher educational level were strongly associated with being at risk of a potential interaction. Factors such as age, race, civil status, citizenship, country of birth, BMI, and physical activity did not show notable associations. Conclusions: Healthcare professionals should be aware of other medical products when they prescribe or dispense a medication or a dietary supplement, especially to the older population and people with a higher educational level.

## 1. Introduction

The use of prescription medicines has increased recently in the United States (US) [1]. This general increase is not homogenous across all drug classes. While important increases are noted in the consumption of some prescription drugs such as anti-hypertensive agents (i.e., angiotensin II receptor blockers and thiazides), consumption of other drug classes, for instance oral antibiotics, has decreased. The use of dietary supplements is high and has remained stable over recent years in the general population in the US. The latest figures point to a prevalence in the use of dietary supplements of around 50% [2]. This use is higher in the older population, and among females, non-Hispanic whites, and people with a higher level of education.

One of the main health concerns related to the use of prescription medicines is the potential risk of adverse events and interactions. Interactions may occur between medications but also between medications and dietary supplements [3]. Qato et al. reported a range of prevalences of concomitant use of medication prescription and dietary supplements of between 0.2% and 2% of the general population [4]. Drug–drug interactions are usually checked by prescription and dispensing systems, which will prompt a warning message to the physician or pharmacist if a drug–drug interaction is detected. However, these systems have no information on patients’ use of dietary supplements, as most of these supplements are obtained over the counter [3].

Interactions between prescription medication and dietary supplements may occur with widely used medications, such as antihypertensive agents and antibiotics [5,6]. The consequences of these interactions may vary between drugs and dietary supplements. Certain minerals including calcium, magnesium, and zinc may interact with tetracycline. The effects of these interactions could cause a reduction in tetracycline absorption (decreasing or eliminating the therapeutic effect) [3,5]. Other minerals, such as potassium, can cause interactions with anti-hypertensive drugs, including angiotensin II receptor blockers, provoking hyperkalaemia [5]. The use of thiazides has to be especially controlled in patients with osteoporosis because they could also interact with vitamin D and/or calcium causing hypercalcaemia and a potential metabolic alkalosis [5].

Since consumption of dietary supplements is rarely supervised by healthcare professionals, this information may help these professionals in their prescription and medication-counselling practice [3]. Specifically, although studies regarding consequences of prescription medication and dietary supplement interaction do exist, only a few of them have focused on American populations [4], revealing the lack of information. Thus, the aim of this paper is to estimate the prevalence of potentially serious drug–dietary supplement interactions among tetracyclines, thiazides, and angiotensin II receptor blocker users by means of the NHANES 2013–2014 dataset. The secondary aim of this paper is to outline the profile of a patient at risk of interactions between medication and dietary supplements. The NHANES survey is among the few large population-based nationally representative health and nutrition studies to apply standard design, and it includes the most detailed information regarding dietary habits and dietary supplement intake, as well as medication prescription. Due to the potential consequences of prescription medication and dietary supplement interaction, it is important to discern what patient profile is at the greatest risk of consuming dietary supplements that cause serious interactions. Thus, the information derived from our study will be important for effective US and international public health planning.

## 2. Methods

### 2.1. Study Design

We obtained data from the 2013–2014 National Health and Nutrition Examination Survey (NHANES). NHNAES is a nationally representative cross-sectional survey conducted by the National Center for Health Statistics (NCHS) and Centers for Disease Control and Prevention. NHANES includes information about the non-institutionalised US population. This database is used worldwide and has produced satisfactory results [6,7].

### 2.2. Ethics

The NCHS Research Ethics Review Board (ERB) approved the 2013–2014 study protocol (protocol 2011-17). NHANES and all participants provided written informed consent.

### 2.3. Interactions under Study

Table 1 shows the drug–dietary supplement interactions studied. We considered medication groups if they represented a prevalence of use higher than 2% of the population included in the database.

Tetracycline interacts with divalent ions such as calcium, magnesium, and zinc, forming a relatively stable and poorly absorbed chelate, preventing absorption of the antibiotic due to a lower amount of calcium in the gut available to be absorbed. This interaction may reduce or even abolish the therapeutic effect of the antibiotic, thereby diminishing anti-infectious efficiency. For this reason, tetracycline should be taken one hour before or two hours after meals [3,5].

Thiazide diuretics can cause increased calcium reabsorption in distal tubules of the kidneys, which contributes to hypercalcemia. Another cause of hypercalcemia is the excess of vitamin D, for example, through high doses of oral supplements, which increases the absorption of calcium in the gut. Due to the retention of calcium in the body, metabolic alkalosis may be developed [5].

Finally, angiotensin II receptor blockers are potassium-sparing and can, therefore, have additional hyperkalaemic effects if combined with potassium supplements or salt substitutes containing potassium. The use of potassium supplements is the main risk factor for developing hyperkalaemia, as this causes a rapid rate of increase in serum potassium levels. Other contributory risk factors such as poor renal function, heart failure, and diabetes should also be considered, as they are associated with a faster rate of hyperkalaemia progress [5,8].

## 3. Population and Prescription Medication Information

The sample was composed of tetracyclines, thiazides, and/or angiotensin II receptor blocker users. These drugs were chosen due to the potential severity of their interactions and their high prevalence of use in the American population. Prescription medication information was obtained through the Prescription Medication subsection included in The Dietary Supplement and Prescription Medication section of the Sample Person Questionnaire. This section provides personal information on the use of prescription medication in the month prior to the participant’s interview. The name of the medication was provided by the participant to the interviewer, who entered it into the computer where it was automatically matched to a generic drug name and code. Medication is presented following the WHO Drug Statistics Methodology of ATC index [9].

*Tetracyclines*: We considered a patient to be a tetracycline consumer if he/she reported having taken a medication with a generic drug name included in the ATC group J01AA.

*Thiazides*: We considered a patient to be a thiazide consumer if he/she reported having taken a medication with a generic drug name included in the ATC group C03AA.

*Angiotensin II receptor blockers*: We considered a patient to be an angiotensin II receptor blocker consumer if he/she reported having taken a medication with a generic drug name included in the ATC group C09CA.

## 4. Dietary Supplement Information

Dietary supplement information was obtained through the dietary supplement subsection also included in The Dietary Supplement and Prescription Medication section of the Sample Person Questionnaire. This subsection allows for collection of personal data on the use of dietary supplements in the month prior to the participant’s interview. Interviewers reported the supplement product name, which was automatically disaggregated to up to 34 nutrients.

We considered a participant to be a nutrient supplement consumer if he/she had taken any supplement containing at least one of the nutrients under study (Table 1).

## 5. Other Covariates

Demographic covariates were sex, age, race/ethnicity (Hispanic origin: Mexican-American, other Hispanic, non-Hispanic white, non-Hispanic black, other race), educational level (primary, secondary, university), civil status (married/with partner, widow/er, divorced, single), citizenship (American/non-American), and country of birth (U.S./Other country).

We also considered body mass index (BMI) and physical activity. BMI was determined from height and weight measured by health technicians previously trained by an expert anthropometrist. We categorised this variable as follows: underweight (<18.5), normal weight (18.5–24.9), overweight (25.0–29.9), and obesity type I (30.0–34.9) and obesity type II and type III (≥35.0).

Physical activity was self-reported and measured through a question asking if participants did any moderate-intensity sports in a typical week. The answer was dichotomic (yes/no).

## 6. Statistical Analysis

Prevalence rates were calculated for each potential interaction described in Table 1. The reference population consisted of those who reported having taken one of the medications under study.

A multivariate logistic regression analysis was conducted to determine the factors associated with a higher probability of having a potential interaction using the presence/absence of the interaction as the dependent variable and demographic and clinical variables as independent variables. The regression model provided odds ratios (OR) and 95% confidence intervals for the associations between the dependent variable and each of the independent variables. Crude associations obtained from bivariate logistic regressions were also presented.

To test potential interactions between demographic and clinical variables, such as age and physical exercise or gender and BMI, we tested the association between the probability of having a potential interaction and the interacting term of the dependent variables. The interacting terms were not statistically significant and, therefore, were not included in the final model.

All analyses were performed taking into account the appropriate weights. This procedure was developed with the goal of obtaining nationally representative estimates and accounting for unequal probability of selection derived from study design and non-response.

STATA 13.1MP was used to perform the statistical analyses.

## 7. Results

### 7.1. Sample Demographic Characteristics

The total sample was made up of 864 prescriptions issued to 820 individuals. Five per cent of those prescriptions involved tetracyclines, 61% involved thiazides, and 46% angiotensin II receptor blockers. Some of these prescriptions consisted of a combination of a thiazide and an angiotensin II inhibitor.

Sociodemographic characteristics are shown in Table 2. The proportion of women was 57%. The majority of the sample was non-Hispanic white (70%) and had secondary-level education (57%). Regarding civil status, 66% of the sample was defined as married or with a partner. Some 88% had been born in the U.S. and a higher percentage (97%) had American citizenship. The main BMI category was type II or III obesity (32%). Only 30% of the sample did moderate-intensity sport.

### 7.2. Prevalence of Potential Interactions

Table 1 shows the prevalence of potential interactions among the sample. Forty-four percent of the people using tetracyclines were consuming calcium and 26% and 37% of them were consuming magnesium and zinc, respectively. Among the users of thiazides, 54% and 52% used calcium and vitamin D, respectively. Finally, 26% of consumers of antagonist II receptor blockers presented a potential interaction due to the concomitant use of potassium. Overall, 49% of the participants were at risk of at least one of the studied interactions.

### 7.3. Factors Associated with Potential Interactions

Table 3 shows the factors associated with suffering a potential interaction between medications and dietary supplements. According to the adjusted analysis, age and educational level were strongly associated with the probability of a potential interaction. Also, compared to the non-Hispanic white population, the non-Hispanic black population presented a lower probability of a potential interaction. The remaining variables (sex, civil status, citizenship, country of birth, BMI category, and physical activity) showed no statistically significant association with the risk of a potential interaction.

Older people had a higher risk of using a prescription medication and a dietary product with a potential interaction effect (OR = 1.02 (95% CI 1.01, 1.03)) per year, i.e., OR is 1.22 in patients 10 years older). People with a higher educational level (secondary or university) showed a higher risk of using a dietary product with a potential interaction with one prescription medicine (OR = 2.0 (95%CI 1.18; 3.23) and OR = 1.6 (95% CI 1.01; 2.63), respectively).

## 8. Discussion

One in every two people who take one of the considered medications is at risk of a potential interaction. Specifically, older people and the population with a higher educational level represent a profile at risk of a potential interaction between medications and nutritional supplements. Older people are also more likely to use both drugs and supplements because of a higher potential to get sick [10]. This is an important issue from a public health perspective, as there are population groups, such as the older population, who are high consumers of these two kinds of health products. This study is one of the first to examine factors associated with specific medication-dietary supplement interactions [11,12].

Qato et al. already showed that more than two-thirds of older adults used prescription medication with OTC medication or dietary supplements [4]. This is in line with our results indicating that the older population has greater probability of suffering a potential interaction. According to Kantor et al. supplement use in the US showed a downward trend among young adults aged 20 to 39 years, stable use among middle-aged adults aged 40–64 years, and an increase among adults over 65 years of age; this last population has a greater probability of being under pharmacotherapeutic treatment due to their clinical status [2]. In this population, the intake of both products is essential. Polypharmacy is a well-known phenomenon that is mainly observed in older populations [13]. The appropriateness of these medications is questioned in some cases [14]; however, in other situations such as antihypertensive or diuretic medication, the need is beyond doubt as cardiovascular illnesses are among the most important causes of death and disability in the US [15]. A similar scenario is observed with dietary supplements, where the effect of the supplementation may reduce the risk of several chronic diseases [16,17].

People with a primary level of education showed lower likelihood of being at risk of a potential interaction. This group also has a lower likelihood of taking dietary supplements. An explanation for this might be that people with a higher educational level might be over-concerned due to a flood of health information about health, and they might be taking dietary supplements when they do not need them. However, this result may also indicate a higher concern regarding the health of people with a higher education; higher education mediates the impact on health outcomes through health literacy [18]. Furthermore, people with a higher education level, which are likely to have a higher socioeconomic status, may have more resources to access dietary products.

These results are especially important for healthcare professionals. Determining whether patients at risk of suffering a potential interaction are taking a dietary supplement that might interact with their prescribed medication is important. In the case of tetracyclines, the effect of the drug may be decreased and the patient may be uncovered for a potential infection; in the case of thiazides, patients may suffer metabolic alkalosis; and in the case of angiotensin II receptor blockers, cardiac function may be affected. This information should be considered at the time of prescription or dispensation. A secondary assessment could be made to analyze whether this supplement is really needed, and if so, to try to adapt it to the pharmacologic treatment. In addition, the assistance of a specialist, such as a nutritionist, is recommendable.

In this line, public policies designed to inform healthcare professionals how to detect potential interactions and tools to help them identify them are highly recommended. These tools could be incorporated in the respective electronic tools of prescription and dispensing, and they might not only remind healthcare professionals to ask about the use of those dietary supplements that may generate an interaction, but also offer alternatives to avoid the interaction in the event that the supplement is recommended. In addition, it is important to inform citizens that, before taking a supplement, it is desirable to consult their doctors to verify that the supplement is necessary and safe taking into account their prescription drugs.

This study has several strengths. It is among the first to evaluate medication use with specific dietary supplements in a representative sample of the American population, providing useful information for targeted public health planning. In the NHANES, medication and dietary supplement use were assessed through in-home interviews, and boxes were seen by the interviewers in most participants. This reduces the recall bias, which is especially notable in medication [1]. However, the present study also has several limitations. First, there is no certainty that the interactions detected occurred. Furthermore, some of these interactions were dose-dependent, and information on dose was not available. Second, information only showed self-reported recent consumption, so it was impossible to discern whether the consumption was concurrent. Finally, other important medication groups that might generate serious interactions, such as quinolones, were not assessed due to their limited representation in the sample. These limitations are not restricted to the present study, as the methodology and analysis strategy followed were similar to those of previous studies based on the NHANES database [4].

## 9. Conclusions

There are two main population groups at risk of potential interactions: older people and the population with a higher educational level. With respect to other races, non-Hispanic whites present a higher risk of potential interactions. These results add important information about ways of approaching patients when they receive a prescription or ask for a medication. Health policy should take this information into account so as to inform healthcare professionals, and electronic tools should be developed or adapted to help in the re-assessment of their pharmacotherapeutic planning.

## Figures and Tables

**Table 1:** Drug–dietary supplement interactions and their consequences.

Prescription Medicine	Dietary Supplement	Potential Clinical Consequences of the Interaction	Prevalence of Potential Interactions
Tetracyclines	Calcium	Decreased therapeutic effect as a consequence of a reduction in the absorption of tetracycline [3].	44.3%
	Magnesium	Decreased therapeutic effect as a consequence of a reduction in the absorption of tetracycline [3,5].	26.9%
	Zinc	Decreased therapeutic effect as a consequence of a reduction in the absorption of tetracycline [3,5].	37.4%
Thiazides	Calcium	Hypercalcemia and metabolic alkalosis. Thiazides reduce the urinary excretion of calcium. Vitamin D increases the absorption of calcium [5].	53.5%
	Vitamin D		52.1%
Angiotensin II receptor blockers	Potassium	Higher risk of hyperkalaemia, especially in patients with decreased renal function, heart failure, or diabetes [5].	28.8%

**Table 2:** Sociodemographic characteristics of the sample (n = 820).

	% or Mean	95% CI
**Gender,% (n)**		
Men	42.6 (350)	38.7; 46.7
Women	57.3 (470)	53.3; 61.3
**Age, mean (range)**	61.4 (20–80)	60.3; 62.6
**Age group,% (n)**		
20–39	6.4 (41)	4.6; 8.8
40–59	34.1 (249)	29.4; 39.2
60–79	50.0 (470)	45.6; 54.4
≥80	9.5 (104)	7.6; 11.9
**Race,% (n)**		
Mexican-American	3.9 (63)	2.0; 7.7
Other Hispanic	3.5 (62)	2.3; 5.5
Non-Hispanic white	70.4 (357)	65.5; 75.0
Non-Hispanic black	15.6 (237)	11.4; 21.0
Other race	6.4 (101)	4.4; 9.2
**Education,% (n)**		
Primary	15.7 (191)	11.9; 20.5
Secondary	57.4 (450)	53.6; 61.1
University	26.9 (179)	22.7; 31.7
Civil status *,% (n)		
Married/with partner	65.5 (482)	62.0; 68.9
Widow/er	12.9 (131)	10.4; 15.8
Divorced	14.4 (131)	12.3; 16.9
Single	7.1 (75)	5.2; 9.5
Citizenship *,% (n)		
American	88.3 (633)	84.4; 91.3
Non-American	11.7 (186)	8.7; 15.6
Country of birth *,% (n)		
U.S.	97.7 (779)	96.1; 98.6
Other country	2.3 (40)	1.4; 3.9
**Body mass index (categories),% (n)**		
Underweight	0.5 (5)	0.1; 2.2
Normal weight	10.6 (109)	7.6; 14.5
Overweight	30.7 (247)	26.4; 35.3
Obesity type I	26.5 (206)	22.2; 31.4
Obesity types II and III	31.6 (253)	28.3; 35.2
**Physical activity in a typical week,% (n)**		
Yes	40.4 (313)	35.9; 45.0
No	59.6 (507)	55.0; 64.1

* The following variables contain one missing value: civil status, citizenship and country of birth.

**Table 3:** Factors associated with potential interactions based on the multivariate weighted logistic regression model ^$^.

	Bivariate Analysis		Multivariate Analysis	
	OR	95% CI	OR	95% CI
**Gender**				
Men	ref		ref	-
Women	1.08	0.79; 1.49	1.23	0.87; 1.75
**Age (1 year increase)**	1.02	1.01; 1.03	1.02	1.01; 1.03
**Race**				
Non-Hispanic white	ref		ref	-
Other Hispanic	0.42	0.17; 1.03	0.44	0.14; 1.33
Mexican-American	0.44	0.24; 0.80	0.55	0.25; 1.20
Non-Hispanic black	0.42	0.29; 0.60	0.45	0.30; 0.66
Other race	0.58	0.83; 1.78	0.60	0.29; 1.26
**Education**				
Primary	ref		ref	-
Secondary	2.10	1.31; 3.40	1.95	1.18; 3.23
University	2.03	1.24; 3.31	1.63	1.01; 2.63
Civil status *				
Married/with partner	ref		ref	-
Widow/er	0.98	0.66; 1.46	0.84	0.50; 1.43
Divorced	0.92	0.65; 1.32	1.05	0.71; 1.56
Single	0.56	0.25; 1.26	0.95	0.39; 2.34
Citizenship *				
American	ref		ref	-
Non-American	0.40	0.16; 0.99	0.86	0.27; 2.72
Country of birth *				
U.S.	ref		ref	-
Other country	0.65	0.41; 1.02	1.12	0.57; 2.20
**Body mass index (categories)**				
Underweight *				
Normal weight	ref		ref	-
Overweight	0.90	0.53; 1.51	0.77	0.45; 1.29
Obesity type I	1.23	0.77; 1.97	1.00	0.59; 1.71
Obesity types II and III	1.17	0.83; 1.67	1.04	0.74; 1.48
**Physical activity in a typical week**				
Yes	ref		ref	-
No	0.69	0.41; 1.17	0.71	0.41; 1.24

^$^ Potential interactions include the interaction of tetracycines with calcium, magnesium or zinc, thiazides with vitamin D and Angiotensin II receptor blockers with potassium; * Few values to be considered. CI = Confidence interval; OR = Odds ratio.
